# Two Cases of Paralysis of the Third Nerve

**Published:** 1871-12

**Authors:** C. K. Fiske

**Affiliations:** St. John, New Brunswick


### TWO CASES OF PARALYSIS OF THE THIRD NERVE.

By C. K. FISKE, M. D., St. John, New Brunswick.

CASE I. Mr. M——, barrister, of this city, aged 40, on the 16th of December, 1869, while driving, incautiously jumped from a sleigh, and coming in collision with another rapidly-driven sleigh, received so severe a concussion as to render him insensible for several hours.

His physician, Dr. Earle, was called, and gave all needed attention to his case till restoration to consciousness, after which he complained of no pain or distress in the head, and there was no external contusion or wound upon the head or body, and he seemed all right with the exception of inability to raise the left eyelid.

On being called to him in consultation, the following day, I found a complete paralysis of the third nerve; closure of the left eyelid, on lifting which, I found the eye turned far to the left and slightly downward, by the combined action of the external rectus and superior oblique muscles; all motion of the eye suspended, the divergence being too great for double vision; the pupil dilated to about mid expansion; the accommodation power suspended, and withal giving the eye a vacant stare; vision confused and uncertain as to distances, causing a staggering gait when attempting to walk. I found the patient in bed, quite comfortable; mind clear and memory good: pulse regular, and skin natural. He was required to remain in bed, and two or three leeches were applied to the temple; and, believing there had been sanguineous effusion near the origin of the nerve, it was decided that rest and rather low diet should be persisted in for a fortnight or more. The Calabar bean was applied to the eye daily, and, after the end of a fortnight, faradization was applied in various ways two or three times a week. Some small blisters were applied over the brow and behind the ear. The bean produced contraction of the pupil to some extent, but the iris did not readily come under its influence.

The patient was allowed to walk about the house and to drive occasionally, and during the month of February warm baths were taken once or twice a week, and iodide of potassium was administered in medium doses three times a day, the battery being applied two or three times a week; also the Calabar bean as at first till the 1st of March, when there was a sudden and marked change in the case. On that day the functions of the nerve became nearly restored, the ptosis entirely relieved, and the inward motion of the eye became nearly perfect; vision improved rapidly, and the accommodation power was soon fully restored, the pupil being contracted to nearly its natural size without the aid of bean.

This case is remarkable for the completeness of the paralysis of the nerve and for the sudden restoration of its conducting power after so long a time—from the 16th of December till the 1st of March following.

Its record may be of interest to the practitioner, and assist in the prognosis of other cases of some weeks' standing, or even months.

This patient had been a "free liver," but readily consented to follow the advice of his physicians, and under a medium diet, with abstinence from stimulants, his general health and tone of body have much improved, and there is very little doubt the final cure was due to these latter means, rather than to the use of the Calabar bean or the battery.

Case II. Mr. T——, of this city, aged 55, of spare habit, but rather full circulation, was suddenly attacked with ptosis of the left eyelid, attended with giddiness, on the 12th of February, 1870.

He was brought immediately to my office, when I found the following appearances: the left eyelid closed, which on lifting by my own hand, I found the pupil largely dilated, and the eyeball turned much to the left and somewhat downward; no inflammation or pain, but he complained of vertigo.

On inquiring into the causes, I learned my patient had been, for many months, smoking large quantities of tobacco; a pipe in his mouth almost at all times, excepting while eating or sleeping. I directed him to go home to his rooms and remain within-doors, to take efficient purgative medicines, and to abstain from smoking.

On the following day I found him much better, vertigo relieved, but with no improvement in the appearance of the eye and lid. I applied the Calabar bean, which readily contracted the pupil, but did not relieve the ptosis I directed iodide of potassium to be taken in three-grain doses three times a day, and abstinence from the use of tobacco in all shapes.

In the course of a fortnight the eye-symptoms were much improved, and by the middle of March the deformity was entirely removed, and at the present date the patient remains quite well.

The improvement in this case was gradual from day to day, while in the first case the change was sudden, after eleven weeks' duration, from complete ptosis, expansion of pupil, and strabismus divergens, to full restoration of the parts.—*N. Y. Med. Journal.*

